# Immune Endotype-Guided Precision Immunotherapy in Sepsis: From Transcriptomics to Bedside Application

**DOI:** 10.7759/cureus.106312

**Published:** 2026-04-02

**Authors:** Olurotimi J Badero, Olutomiwa Omokore, Ojeyemi Oore-ofe, Bamikole Osibowale, Ibrahim Quadri, Temiloluwa Olayinka, Deborah Olabode, Karen E Kalu, Adeyemi Adetola, Mariam O Buari, Iyiola O Solanke

**Affiliations:** 1 Interventional Cardiology, Iwosan Lagoon Hospital, Lagos, NGA; 2 Interventional Cardiology, Division of Cardio-Nephrology, Cardiac Renal & Vascular Associates, Jackson, USA; 3 Internal Medicine, Babcock University Teaching Hospital, Ilishan-Remo, NGA; 4 Internal Medicine, Benjamin S. Carson College of Health and Medical Sciences, Ilishan-Remo, NGA; 5 General Practice, Lagoon Hospital, Lagos, NGA; 6 Cardiology, Caribbean Tristate Heart Institute, Trinidad, TTO; 7 Surgery, Babcock University, Ilishan-Remo, NGA; 8 Surgery, General Hospital Lagos, Lagos, NGA; 9 Medicine, University of Nigeria, Nsukka, NGA; 10 Medicine and Surgery, Lagos State University College of Medicine, Ikeja, NGA; 11 Medicine, Babcock University Teaching Hospital, Ilishan-Remo, NGA

**Keywords:** biomarkers, critical care, hyperinflammation, immune endotypes, immunoparalysis, immunotherapy, precision medicine, sepsis

## Abstract

Sepsis is a life-threatening syndrome characterized by profound biological heterogeneity and dynamic immune dysregulation. Traditional uniform treatment strategies have failed to account for distinct immune endotypes ranging from hyperinflammation to immunoparalysis. Advances in transcriptomic, proteomic, and cellular profiling have enabled the identification of reproducible immune phenotypes with differing prognoses and therapeutic responsiveness. Hyperinflammatory states may benefit from targeted cytokine inhibition, whereas immunosuppressed phenotypes marked by reduced monocyte HLA-DR (human leukocyte antigen-DR) expression, lymphopenia, and T-cell exhaustion may require immunostimulatory therapies such as GM-CSF (granulocyte-colony stimulating factor), interferon-γ, or interleukin-7. Emerging evidence suggests that aligning immunomodulatory interventions with the patient’s prevailing immune profile could improve outcomes and avoid harm associated with non-stratified therapy. This study was conducted as a structured narrative scoping review, with a comprehensive literature search of PubMed/MEDLINE, Embase, Scopus, and Web of Science covering publications from 2010 to 2025, using keywords related to sepsis, immune dysregulation, endotypes, biomarkers, and precision medicine. This review aims to examine the role of immune endotypes in the pathophysiology and clinical heterogeneity of sepsis and to evaluate emerging evidence on biomarker- and transcriptomic-guided precision immunotherapy. Implementation of precision medicine in sepsis depends on standardized endotyping tools, serial immune monitoring, and biomarker-enriched adaptive trial designs. Although logistical and translational barriers remain, immune-guided therapy represents a promising but unproven paradigm that requires rigorous prospective validation through adaptive, biomarker-enriched trials.

## Introduction and background

Sepsis is a major global health crisis, responsible for or contributing to approximately one in five deaths worldwide. An estimated 49 million cases occur each year, leading to about 11 million deaths annually. Despite significant advances in antibiotic therapy and supportive care, sepsis continues to pose a serious challenge in intensive care units around the world [1,2].

Limitations of the “one-size-fits-all” approach to sepsis management

To facilitate early recognition and timely initiation of therapy, several diagnostic criteria have been established for sepsis. These include the Systemic Inflammatory Response Syndrome (SIRS) criteria, the Sequential Organ Failure Assessment (SOFA) criteria, and clinical assessment frameworks based on patients’ physiological responses and the presence and extent of organ dysfunction, respectively [2].

Despite the application of established clinical criteria for the early recognition and management of sepsis, marked interindividual variability in treatment response and clinical outcomes persists. It is critical to note that numerous large Phase III trials of immunomodulators, including anti-TNF and anti-IL-1 therapies, failed over the past three decades, largely due to the enrollment of biologically heterogeneous sepsis populations without stratification, thereby masking potential benefits in responsive subgroups. This historical limitation underpins the current shift toward immune endotyping and precision-guided therapy [3]. Such heterogeneity is largely attributable to distinct host immune responses at the molecular level to invading pathogens and their associated products. Consequently, sepsis has been increasingly characterized into distinct immunologic and molecular endotypes [3,4].

Reliance solely on conventional clinical parameters for the diagnosis, management, and prognostication of sepsis is therefore becoming increasingly limited. Future clinical practice is expected to incorporate endotype-based stratification to enable more precise and individualized therapeutic approaches [5].

Emergence of immune dysregulation as a central driver of outcomes

The immune response and its regulatory mechanisms constitute essential host defense processes. However, when dysregulated, these protective mechanisms may become maladaptive and contribute to tissue injury and organ dysfunction. Indeed, the clinical and biochemical manifestations of sepsis are largely a consequence of the host immune response rather than the pathogen itself [6].

Historically, sepsis was conceptualized as a unidirectional hyperinflammatory syndrome that progressed to a subsequent state of immunoparalysis. This paradigm has been substantially revised. Contemporary evidence indicates that, at the onset of septic shock, patients stratify into distinct immune activation states. Some exhibit exaggerated proinflammatory responses, others demonstrate profound anti-inflammatory immunosuppression, and many present with overlapping or mixed endotypes. These divergent immune trajectories are closely associated with clinical outcomes.

Hyperinflammatory endotypes, including macrophage activation syndrome and interferon-γ-driven sepsis, are associated with increased early mortality. Conversely, sepsis-induced immunoparalysis, characterized by marked downregulation of monocyte human leukocyte antigen-DR (HLA-DR) expression, confers an elevated risk of secondary infections and late mortality. Collectively, these findings underscore that immune dysregulation, rather than infection alone, constitutes a principal determinant of prognosis in sepsis [7].

Rationale for focusing on immunoparalysis, sepsis endotypes, and precision medicine

The recognition of discrete immune endotypes provides a strong rationale for precision medicine in sepsis. Sepsis-induced immunosuppression (SII), present in up to 40% of patients and defined by markedly reduced monocyte HLA-DR expression, represents a quantifiable and clinically actionable state of immune failure. Similarly, proinflammatory endotypes, such as macrophage activation-like syndrome (MALS) and interferon-driven sepsis (IDS), can be identified using protein biomarkers, including ferritin and CXCL9, enabling targeted enrollment into immunomodulatory trials [7].

While genomic endotyping systems further refine risk stratification, their cost, complexity, and temporal instability limit bedside applicability. In contrast, biomarker-guided immune profiling offers a rapid, scalable approach to tailor therapy, minimize mistimed interventions, and align treatment with the patient’s prevailing immune state [7].

The aim of this review is to synthesize current evidence on immune endotypes in sepsis and explore how transcriptomic, biomarker, and immunologic profiling can inform precision immunotherapy. It also seeks to assess the clinical potential and challenges associated with translating immune endotype-guided treatment strategies into bedside practice in critical care medicine.

## Review

Methodology

This study was conducted as a structured narrative scoping review aimed at synthesizing current evidence on sepsis endotypes and their application in precision immunotherapy. A comprehensive literature search was performed using PubMed/MEDLINE, Embase, Scopus, and Web of Science, covering publications from 2010 to 2025, with foundational mechanistic studies included where relevant. Search terms combined keywords related to sepsis, immune dysregulation, endotypes, transcriptomics, biomarkers, immunoparalysis, immunotherapy, and precision medicine.

Eligible studies included randomized controlled trials, cohort studies, translational research, and high-quality reviews evaluating biological endotype classification, biomarker-guided treatment strategies, or associations between immune profiles and outcomes in adult or pediatric sepsis. Articles were screened by title, abstract, and full text, and data were extracted on study design, endotype identification methods, therapeutic interventions, and outcomes. Due to methodological heterogeneity, findings were synthesized narratively and organized into key domains, including immune mechanisms, omics-based stratification, biomarker-guided therapy, timing of immunomodulation, and implementation challenges.

Sepsis as a spectrum of immune dysregulation

Sepsis is a dynamic syndrome in which proinflammatory and immunosuppressive pathways coexist, with their relative predominance shaping the clinical course [8]. In the early phase, hyperinflammation predominates, driven by excessive cytokine release, endothelial activation, and multiorgan dysfunction, manifesting clinically as fever, hemodynamic instability, and elevated acute-phase reactants [8,9].

Among survivors of the initial insult, the immune response frequently transitions toward sustained immunosuppression [8]. This phase is characterized by persistent lymphopenia, diminished ex vivo TNF production, reduced monocyte HLA-DR expression, and increased susceptibility to secondary infections and viral reactivation [10]. Mechanistically, this reflects defects in both innate and adaptive immunity, including apoptosis of immune cells, expansion of regulatory T cells, T-cell exhaustion, accumulation of myeloid-derived suppressor cells, and impaired co-stimulatory signaling [11].

Rather than a simple biphasic model, contemporary evidence supports simultaneous activation of pro- and anti-inflammatory pathways. Inhibitory checkpoints such as programmed cell death protein 1 (PD-1) and cytotoxic T lymphocyte-associated protein 4 (CTLA-4) are upregulated, anti-inflammatory cytokines, including IL-10 and TGF-β, predominate, and antigen presentation is impaired due to HLA-DR downregulation [8,12-15]. Collectively, these processes culminate in immunoparalysis.

In the early phase, pathogen recognition via toll-like receptors (TLRs) on innate immune cells induces a cytokine storm. Simultaneously, counter-regulatory mechanisms are initiated. As sepsis progresses, the immune system undergoes dynamic reprogramming rather than uniform suppression. This includes regulatory T-cell (Treg) expansion, persistent expression of inhibitory checkpoints such as PD-1 and CTLA-4, and extensive apoptosis of T and B lymphocytes, ultimately reducing adaptive competence [16-18].

This immunological dysfunction observed in sepsis involves both adaptive and innate compartments, impairing T-cell receptor (TCR) signaling, suppressing cytokine production (e.g., IL-2, IFN-γ), and limiting cytotoxic activity, resulting in a dysfunctional adaptive landscape [10,19].

Innate dysfunction is further amplified by aberrant activation of the cGAS-STING pathway triggered by mitochondrial DNA accumulation, promoting type I interferon and IL-10 production via JAK-STAT3 signaling [20-22]. Dendritic cell apoptosis, reduced co-stimulatory molecule expression, and impaired cytokine production further compromise host defense [9,11,22]. The result is a fluctuating and heterogeneous immune landscape that directly influences outcomes.

Sepsis phenotypes and endotypes

Clinical Phenotypes

Clinical phenotyping traditionally relies on bedside variables, organ dysfunction patterns, laboratory abnormalities, and illness trajectories. Large cohort analyses have identified reproducible clinical clusters differing in shock severity, inflammatory markers, coagulopathy, and mortality risk [23]. These phenotypes provide pragmatic tools for early risk stratification.

However, clinical clusters reflect downstream manifestations rather than underlying biology. Patients with similar physiologic profiles may exhibit divergent immune responses at the molecular level, resulting in variable treatment responsiveness [24]. Furthermore, clinical phenotypes are often static and may not capture temporal immune evolution. Thus, while operationally useful, they lack mechanistic specificity for precision immunotherapy.

Biological Endotypes

Biological endotypes define sepsis subgroups based on shared molecular and immunologic mechanisms. Transcriptomic profiling has identified reproducible gene-expression signatures linked to immune activation, interferon signaling, antigen presentation pathways, and T-cell exhaustion [24,25]. Proteomic and cellular analyses further distinguish inflammatory mediator patterns and alterations in monocyte HLA-DR expression and lymphocyte subsets.

A key distinction exists between hyperinflammatory endotypes, characterized by excessive cytokine signaling and endothelial injury, and immunosuppressed endotypes, marked by lymphopenia, impaired antigen presentation, and increased risk of infection [26]. These states may coexist or evolve sequentially.

Pediatric septic shock endotypes have shown relative reproducibility and associations with corticosteroid responsiveness [27]. Adult endotypes demonstrate greater heterogeneity and temporal instability influenced by age, comorbidities, and pathogen diversity [24]. This dynamic behavior highlights the need for serial immune monitoring rather than one-time classification.

Relevance of Endotyping to Outcomes and Therapy

Endotypes carry important prognostic implications. Hyperinflammatory signatures correlate with early organ dysfunction and short-term mortality, whereas immunosuppressive profiles are associated with prolonged ICU stay and secondary infections [3,4]. Gene-expression risk models provide prognostic information beyond conventional severity scores [2].

Emerging evidence suggests that biological endotypes may predict treatment responsiveness. Corticosteroid therapy, IL-1 blockade, and immunostimulatory strategies appear to demonstrate differential effects depending on immune profile [3,5]. These findings support the integration of biological endotyping into therapeutic decision-making, though assay standardization and temporal variability remain barriers to widespread adoption.

Precision medicine in sepsis: cutting-edge approaches and personalized care

Moving From Syndromic to Biology-Driven Management

Conventional sepsis management emphasizes antimicrobial therapy, hemodynamic resuscitation, and organ support without addressing host-specific immune responses. Despite numerous randomized trials, major outcome improvements remain limited [28]. Incorporating immunologic profiling and endotype classification offers a pathway toward targeted, individualized interventions [5].

Lessons From Oncology and Immunology

Precision medicine in sepsis draws from oncology and immunology, where biomarker-defined therapy has transformed outcomes. Targeted treatments such as HER2-directed therapy in breast cancer, EGFR inhibition in lung cancer, and PD-1 blockade in melanoma demonstrate the value of genotype- or immune-driven treatment allocation [29-31]. Similarly, TNF inhibitors in rheumatoid arthritis and IL-5 antagonists in eosinophilic asthma illustrate how immune phenotyping guides biologic therapy [32,33].

Transcriptomic profiling in sepsis has identified reproducible host-response endotypes [3,34], and early biomarker-guided interventions, including IL-1 blockade in hyperinflammatory phenotypes, suggest improved organ dysfunction outcomes compared with non-selective therapy [7]. As in oncology, effective sepsis management will likely depend on identifying biologically coherent subgroups rather than applying uniform protocols to a heterogeneous syndrome.

Omic-Based and Computational Tools

High-throughput genomics, transcriptomics, metabolomics, and immune profiling enable multidimensional characterization of host-pathogen interactions. Whole-blood transcriptomic analyses have consistently identified endotypes associated with mortality and organ dysfunction [3,34]. Metabolomic and immune phenotyping approaches further delineate bioenergetic failure and immune exhaustion states.

Machine learning and artificial intelligence facilitate the interpretation of high-dimensional datasets. Unsupervised clustering defines biologically coherent endotypes, while supervised models enhance the prediction of mortality and treatment response [3,24]. Integration of omics data with clinical variables improves prognostic accuracy beyond conventional scoring systems.

The ultimate goal is real-time integration into ICU workflows. Rapid molecular diagnostics and AI-enabled decision-support tools could match patients to targeted therapies. However, challenges, including assay standardization, validation across populations, model interpretability, and demonstrating improved outcomes, remain [2,7].

Biomarker-Guided Immunotherapy

SII can be identified using cellular, soluble, and functional biomarkers. Reduced monocyte HLA-DR expression and persistent lymphopenia correlate with adverse outcomes and have been used to stratify patients for immunostimulatory therapies [9]. Elevated IL-10, IL-6, and checkpoint molecule expression, such as PD-1, reflect immune dysregulation [35]. Functional assays, including reduced ex vivo TNF-α production, directly assess immune responsiveness [36].

While promising, biomarkers lack universally accepted thresholds and may fluctuate over time. Composite panels or transcriptomic signatures may offer superior stratification compared with single markers [9,35].

Immunostimulatory Therapies

GM-CSF (granulocyte-colony stimulating factor), interferon-γ, IL-7, and immune checkpoint inhibitors have been investigated to reverse SII. GM-CSF increases mHLA-DR expression and may reduce secondary infections [36]. Interferon-γ restores macrophage activation [37], and IL-7 reverses lymphopenia with favorable immunologic endpoints [38]. Early exploration of PD-1/PD-L1 blockade mirrors oncology approaches [35]. Myeloid-derived suppressor cell (MDSC)-targeted therapies represent an emerging strategy to reverse immune suppression in conditions such as sepsis and cancer. MDSCs are a heterogeneous population of immature myeloid cells that expand during chronic inflammation and contribute to T-cell dysfunction through mechanisms such as arginase-1 activity, nitric oxide production, and immune checkpoint expression. Therapeutic strategies include depletion of MDSCs, inhibition of their suppressive function, blockade of their recruitment, and promotion of their differentiation into mature myeloid cells [39].

Evidence suggests that biomarker-guided strategies improve organ dysfunction scores in selected patients [40]. However, safety concerns remain, as immunostimulation in hyperinflammatory states may worsen organ injury. Larger trials are needed to confirm survival benefits.

Immunosuppressive and Immunomodulatory Therapies

Corticosteroids remain widely studied, shortening shock duration and possibly reducing mortality in selected vasopressor-dependent patients [41,42]. IL-1 receptor antagonism has shown benefit in macrophage activation-like phenotypes [43], whereas broad TNF inhibition has largely failed in unselected populations [44]. Extracorporeal cytokine removal devices reduce circulating inflammatory mediators to help restore immune balance, with some observational evidence showing decreased cytokine levels and vasopressor needs. However, strong randomized evidence for survival benefit is limited, and effectiveness likely depends on appropriate patient selection and timing [45].

These therapies underscore the principle of endotype-specific effectiveness, as immunosuppression may harm patients already immunoparalyzed [24].

Timing of Immunostimulation Versus Immunosuppression

Sepsis represents a dynamic continuum in which hyperinflammation and immunosuppression may coexist [9]. Mistimed immunomodulation risks harm: immunosuppressive therapy in immunoparalyzed patients may increase infection risk, whereas immunostimulation during uncontrolled hyperinflammation may worsen organ failure [36,37].

Dynamic immune monitoring, including serial mHLA-DR measurement and transcriptomic reassessment, supports the concept of immune phase-adapted therapy, aligning intervention with the dominant immune phenotype at a given time point [36,38].

Knowledge gap and research priorities

Major gaps include a lack of standardized endotype definitions, assay variability, and limited bedside applicability of advanced profiling [24,46]. Adaptive, biomarker-enriched trial designs are needed to test targeted interventions [40]. Equitable implementation in low- and middle-income countries remains essential to avoid widening global disparities [47].

Implications for clinical practice and future directions

Practical implementation requires simplified assays embedded within ICU workflows and linked to decision-support algorithms [24,45]. Biological stratification may reduce therapeutic heterogeneity, improve organ dysfunction trajectories, and minimize harm from inappropriate immunomodulation [24,40].

The path forward requires consensus definitions, standardized biomarkers, adaptive trials, and equitable access. Ultimately, precision sepsis care depends not only on identifying the correct therapy but also on delivering it at the biologically appropriate time.

## Conclusions

Sepsis is increasingly recognized as a biologically heterogeneous and dynamically evolving syndrome of immune dysregulation rather than a uniform hyperinflammatory condition amenable to standardized treatment. Advances in transcriptomic, proteomic, and cellular profiling have identified reproducible immune endotypes ranging from hyperinflammatory states to immunoparalysis, each associated with distinct prognoses and therapeutic responses. Emerging evidence indicates that immunomodulatory therapies must be matched to the patient’s prevailing immune phenotype, targeted cytokine blockade for selected hyperinflammatory profiles, and immunostimulatory approaches for immunosuppressed states, highlighting the limitations of non-stratified interventions. Achieving precision medicine in sepsis requires standardized immune endotyping, serial monitoring to capture temporal changes, and adaptive, biomarker-enriched clinical trials supported by omics technologies and computational tools. Although practical and equity challenges remain, endotype-guided therapy represents a potential paradigm shift toward delivering the right immunomodulatory treatment to the right patient at the appropriate time.
